# Enlargement of choroid plexus in complex regional pain syndrome

**DOI:** 10.1038/srep14329

**Published:** 2015-09-21

**Authors:** Guangyu Zhou, Jaakko Hotta, Maria K. Lehtinen, Nina Forss, Riitta Hari

**Affiliations:** 1 Department of Neuroscience and Biomedical Engineering, Aalto University, FI-00076 AALTO, Espoo 02150, Finland; 2 Aalto NeuroImaging, Aalto University, Espoo 02150, Finland; 3 Clinical Neurosciences, Neurology, University of Helsinki and Helsinki University Hospital, Helsinki 00290, Finland; 4 Department of Pathology, Boston Children’s Hospital, Harvard Medical School, Boston, Massachusetts 02115, USA

## Abstract

The choroid plexus, located in brain ventricles, has received surprisingly little attention in clinical neuroscience. In morphometric brain analysis, we serendipitously found a 21% increase in choroid plexus volume in 12 patients suffering from complex regional pain syndrome (CRPS) compared with age- and gender-matched healthy subjects. No enlargement was observed in a group of 8 patients suffering from chronic pain of other etiologies. Our findings suggest involvement of the choroid plexus in the pathogenesis of CRPS. Since the choroid plexus can mediate interaction between peripheral and brain inflammation, our findings pinpoint the choroid plexus as an important target for future research of central pain mechanisms.

The choroid plexus is the key producer of cerebrospinal fluid, which provides a fluid cushion for the central nervous system and a sink for nervous-system biomarkers and debris^1^,^2^. The choroid plexus also secretes into the cerebrospinal fluid a wide array of proteins and other signaling substances that instruct the development and maintenance of the mammalian brain^2^. Moreover, the choroid plexus provides a point of entry for immune cells into the brain, thereby linking the peripheral and central inflammatory systems^3^,^4^.

Until now, the choroid plexus has been largely neglected in clinical neuroscience and its potential role in neurological disorders has remained unresolved^5^. For example in human brain imaging studies, choroid plexus volume is typically not discussed or quantified with a few exceptions^6^,^7^,^8^,^9^. The normal-size criteria for the pediatric population were only recently proposed^10^, and similar criteria for the adult choroid plexus remain to be established.

Here, we report an unexpected association between choroid plexus volume and central pain. Using MRI, we found choroid plexus enlargement in patients suffering from complex regional pain syndrome (CRPS), a chronic and multifactorial pain disorder that initially follows a minor peripheral trauma and subsequently spreads to areas beyond the original lesion area^11^,^12^. The mechanisms underlying the CRPS evolvement are still poorly understood, and thus our findings implicating the involvement of choroid plexus in CRPS could elucidate the pathogenesis of this debilitating condition.

## Results

**Figure 1:**
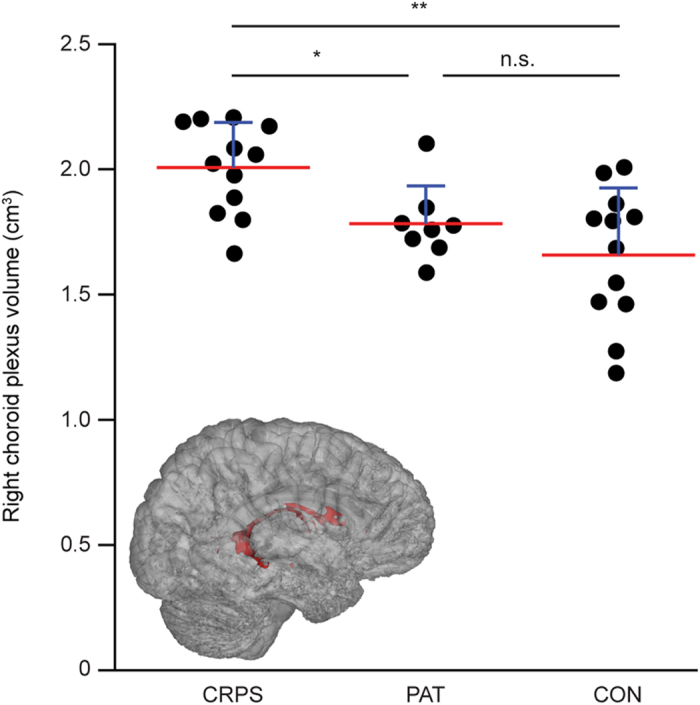
Volumes and 3D rendering of the right lateral-ventricle choroid plexus. The scatter plot shows volumes of the right lateral-ventricle choroid plexus in CRPS patients (CRPS), pain patients suffering from chronic pain of other etiologies (PAT), and controls subjects (CON). **Bonferroni-corrected p = 0.0073 in two-tailed Mann-Whitney U test, *p = 0.045, n.s. p = 1.69. Red lines = mean, blue lines = standard deviation. The insert shows a 3D model of the right lateral-ventricle choroid plexus (shown in red) of one CRPS patient, which was created using FSLView’s 3D Rendering.

We performed MRI image analysis of 29 distinct subcortical brain regions in CRPS patients and healthy control subjects to identify if changes in these regions correlated with CRPS. Image analysis from a cohort of 12 CRPS type 1 female patients revealed a striking increase in the volume of the right lateral-ventricle choroid plexus. Figure 1 shows group-wise volumes of the right lateral-ventricle choroid plexus, with a statistically significant group effect (F(2, 29) = 8.31, effect size η^2^ = 0.36, FDR-adjusted p = 0.041). The right lateral-ventricle choroid plexus was 21.1% larger in the CRPS patients compared with the healthy control subjects (effect size Cohen’s d = 1.53, corrected p = 0.0073), and 12.6% larger than in the other control group consisting of patients suffering from chronic pain of other etiologies (d = 1.35, corrected p = 0.045). We also found a large group effect on the volumes of the left lateral ventricle (F(2,29) = 6.84, η^2^ = 0.32, uncorrected p = 0.0037, FDR-adjusted p = 0.054) and the right thalamus (F(2,29) = 5.46, η^2^ = 0.27, uncorrected p = 0.0097, FDR-adjusted p = 0.094). Patients with CRPS had a larger left lateral ventricle (d = 1.48, corrected p = 0.011) and smaller right thalamus (d = 1.23, corrected p = 0.024) than the healthy control subjects. No statistically significant group effects were observed for any of the other structures examined (all uncorrected p > 0.05, Table 1). No statistically significant correlation was observed between CRPS duration and the volume of the right lateral-ventricle choroid plexus, left lateral ventricle, or right thalamus (all p > 0.5).

## Discussion

We report an unexpected enlargement of the choroid plexus in CRPS patients compared with age- and gender-matched healthy control subjects. While the choroid plexus did not belong to our initial list of structures anticipated to be involved in CRPS, the observed volume increase of 21.1% raises the possibility that the choroid plexus plays a role in the pathogenesis of CRPS.

Previous studies have reported gray-matter changes in CRPS but have not examined the choroid plexus^13^,^14^,^15^,^16^. These studies used voxel-based morphometry analysis of brain structures, and although they addressed subcortical structures as well, they failed to detect abnormalities in e.g. thalamus^14^,^15^,^16^. A key difference between our study and previous reports is the analysis method. We analyzed group differences in brain structures at volume rather than voxel level, and these approaches can yield distinct results^17^. Our approach also uncovered a previously unknown change in ventricular volume in CRPS patients, which again may have been facilitated by our approach to analyze hemisphere-specific structural changes rather than combining total ventricular volume.

While our study identified increased volumes of the CRPS patients’ right lateral-ventricle choroid plexus, right thalamus, and left lateral ventricle, the mechanisms underlying these structural changes remain unknown. The increased volume of the choroid plexus, for example, could result from the concurrent activation of several biological processes including increased proliferation of choroid-plexus cells (as has been shown in cases of direct tissue injury^18^), invasion of the choroid plexus by non-resident immune cells, and/or edema. Future studies will be required to elucidate the precise physiological processes and mechanisms regulating structural changes in CRPS.

Growing evidence suggests a role for the choroid plexus in the pathophysiology of pain. For example, the choroid plexus is an important mediator between brain and peripheral inflammation (for a review, see ref. 3), and recent findings suggest an association between brain inflammation and chronic pain^19^. Analyses of blood and cerebrospinal fluid samples from CRPS patients have revealed increased levels of cytokines^20^. Together with our observations that the choroid plexus is enlarged in CRPS, these findings support contribution of central and peripheral inflammation to the pathophysiology of CRPS.

Another association between choroid plexus and pain stems from Aquaporin 1 (AQP-1), a hydrophobic water-transporting protein expressed at the plasma membrane of choroid plexus epithelial cells^21^. AQP-1 tunes pain perception in mouse dorsal root ganglia^22^ where AQP-1 is upregulated following peripheral nerve injury or trauma. In the dorsal column, AQP-1 has a dual role in the transmission of noxious stimuli as well as in regulating axonal growth. Since AQP-1 is highly expressed by choroid plexus epithelial cells, these findings raise a possibility that AQP-1-targeted treatments might provide a future therapeutic avenue for CRPS^23^. Moreover, the choroid plexus secretes hundreds of factors into the cerebrospinal fluid, including proenkephalin^24^, an endogenous opioid polypeptide hormone. Choroid plexus-proenkephalin is highly expressed and secreted during brain development, and this process persists, albeit at lower intensity in adulthood. While the site(s) of action and role(s) for choroid-plexus-secreted proenkephalin remain to be identified, these findings suggest that the choroid plexus may be involved in mediating responses to pain.

In summary, our results demonstrate an earlier-unexplored association between the choroid plexus and central pain. As limitations of our study we acknowledge the small sample size that resulted in limited statistical power, with the risk for type I errors despite the large effect size. Moreover, while all of our subjects suffered from right-sided pain, our data do not explain the right-hemisphere lateralization of the effect. However, our findings suggest that quantifying choroid plexus volume by contrast-enhanced MRI in future studies with larger cohorts could improve our understanding of the pathophysiology underlying CRPS. Finally, pairing these studies with animal models of CRPS could open avenues for developing improved diagnostics and therapeutic approaches for CRPS.

## Methods

This study was approved by the ethics committee of the Helsinki and Uusimaa Hospital District. Subjects signed informed consent before participation, and all methods were carried out in accordance with the approved guidelines.

### Subjects

We analyzed the structural MRIs of 32 right-handed subjects: (1) twelve CRPS type 1 patients (all females, ages 36–58 years, mean 46; all with right-sided pain; mean disease duration ± SD: 5.8 ± 4.5 years, range 1.4–15.5 years), (2) eight chronic pain patients of other etiologies (2 females, 6 males; ages 38–72 years, mean 54; mean disease duration: 8.5 ± 10.1 years, range 1–30 years), and (3) twelve healthy control subjects (all females, ages 25–60 years, mean 45).

During subject recruitment, CRPS diagnosis was confirmed by clinical examination performed by an experienced neurologist at the Pain Clinic of the Helsinki University Hospital. Eleven patients fulfilled the “research criteria” for CRPS, and the remaining patient fulfilled the less stringent “clinical criteria” for CRPS^25^. None of the patients showed electroneuromyographic signs of major nerve injury, thereby agreeing with the diagnosis of CRPS type 1.

Prior to MRI measurements on the test day, CRPS patients reported a pre-MRI mean pain intensity of 5.5 ± 1.6 (range 3–7) on scale from 0 (no pain) to 10 (maximum pain), and the patients of the control group reported a mean pain intensity of 5.8 ± 1.8 (range 3–8). Neither disease duration nor pain intensity were found to differ between the two patient groups (p = 0.97 and p = 0.88, respectively, two-tailed Mann-Whitney U test).

### Brain MRI

We acquired high-resolution 1 × 1 × 1 mm^3^ T1-weighted MR images at the Advanced Magnetic Imaging Centre of Aalto NeuroImaging, Aalto University. Nine CRPS patients, eight control pain patients, and nine healthy control subjects were scanned with a Signa 3T scanner (model EXCITE for the control pain patients and HDxt for the others; GE Healthcare, Milwaukee, Wisconsin) with a 16-channel head coil. Four CRPS patients and four control subjects were scanned with a Magnetom Skyra 3T scanner (Siemens Healthcare, Erlangen, Germany) with a 30-channel head coil.

Images were acquired with ultrafast gradient-echo 3D sequences (3D fast SPGR with GE scanner, MPRAGE with Siemens scanner) with 176 sagittal slices and a matrix size of 256 × 256. For the CRPS patients and healthy control subjects, the other imaging parameters were TR 10 ms/2530 ms, TE 2.9 ms/3.3 ms, flip angle 15°/7° for GE/Siemens scanner. For the control pain patients (GE scanner) the corresponding parameters were TR 9 ms, TE 1.9 ms, FA 15°.

### Image Analysis

Volumetric segmentation of the MRI images was performed in a standard manner using Freesurfer image analysis suite (http://surfer.nmr.mgh.harvard.edu). The processing included motion correction, removal of non-brain tissue using a hybrid watershed/surface deformation procedure^26^, automated Talairach transformation, and segmentation of the subcortical white matter and deep gray matter volumetric structures^27^,^28^. We analyzed the volumes of 29 subcortical structures (Table 1).

### Statistical Analysis

The MRI data were first adjusted for confounding factors including gender, age, scanner, and total intracranial volume using linear regression method. We conducted a one-way analysis of variance to compare the group effect on the 29 volumes. Multiple comparisons were corrected using the false discovery rate (FDR) controlling procedure^29^. Post-hoc analysis was performed with three pair-wise, independent, two-tailed Mann-Whitney U tests, followed by Bonferroni correction.

We calculated the Pearson correlation (covariates: age, scanner, and total intracranial volume) between disease duration and all brain volumes where our previous analysis had indicated a statistically significant group effect in the CRPS cohort.

## Additional Information

**How to cite this article**: Zhou, G. *et al*. Enlargement of choroid plexus in complex regional pain syndrome. *Sci. Rep*. **5**, 14329; doi: 10.1038/srep14329 (2015).

## Figures and Tables

**Table 1:** Volumetric analysis of variance results for all 29 Freesurfer derived subcortical structures.

Region		CRPS (cm^3^)	PAT (cm^3^)	CON (cm^3^)	F(2,29)	P value	η^2^	FDRadjusted
Choroid Plexus	R	2.01 ± 0.18	1.78 ± 0.15	1.66 ± 0.27	8.31	0.0014	0.364	0.041
	L	1.68 ± 0.29	1.60 ± 0.10	1.51 ± 0.24	1.58	0.2229	0.098	0.714
Lateral Ventricle	R	9.30 ± 3.24	7.94 ± 2.67	7.25 ± 2.87	1.47	0.2467	0.092	0.714
	L	10.89 ± 3.05	8.58 ± 2.60	6.78 ± 2.45	6.84	0.0037	0.320	0.054
Inferior Lateral Ventricle	R	0.35 ± 0.13	0.34 ± 0.13	0.31 ± 0.14	0.30	0.7459	0.020	0.901
	L	0.48 ± 0.14	0.41 ± 0.26	0.37 ± 0.11	1.22	0.3111	0.077	0.714
Third Ventricle		1.10 ± 0.22	0.97 ± 0.43	0.96 ± 0.20	0.88	0.4249	0.057	0.817
Fourth Ventricle		1.55 ± 0.29	1.49 ± 0.25	1.54 ± 0.32	0.11	0.8946	0.008	0.977
Thalamus	R	6.10 ± 0.43	6.27 ± 0.49	6.89 ± 0.80	5.46	0.0097	0.274	0.094
	L	6.24 ± 0.48	6.40 ± 0.59	6.89 ± 0.93	2.64	0.0883	0.154	0.512
Hippocampus	R	4.17 ± 0.29	4.17 ± 0.47	4.32 ± 0.37	0.61	0.5518	0.040	0.842
	L	3.84 ± 0.29	3.87 ± 0.46	4.03 ± 0.31	1.07	0.3566	0.069	0.739
Caudate	R	3.57 ± 0.32	3.58 ± 0.57	3.69 ± 0.36	0.30	0.7448	0.020	0.901
	L	3.41 ± 0.32	3.48 ± 0.49	3.67 ± 0.34	1.60	0.2185	0.100	0.714
Putamen	R	5.31 ± 0.58	5.38 ± 0.40	5.47 ± 0.32	0.35	0.7053	0.024	0.901
	L	5.77 ± 0.53	5.85 ± 0.69	5.81 ± 0.57	0.05	0.9520	0.003	0.977
Pallidum	R	1.50 ± 0.09	1.51 ± 0.24	1.56 ± 0.21	0.46	0.6372	0.031	0.880
	L	1.59 ± 0.17	1.55 ± 0.27	1.67 ± 0.27	0.68	0.5144	0.045	0.829
Amygdala	R	1.85 ± 0.23	1.82 ± 0.43	1.85 ± 0.26	0.03	0.9749	0.002	0.977
	L	1.64 ± 0.18	1.62 ± 0.42	1.62 ± 0.20	0.02	0.9770	0.002	0.977
Accumbens Area	R	0.69 ± 0.10	0.73 ± 0.13	0.71 ± 0.12	0.23	0.7953	0.016	0.923
	L	0.63 ± 0.10	0.66 ± 0.12	0.68 ± 0.16	0.49	0.6197	0.032	0.880
Ventral Diencephaion	R	3.81 ± 0.17	3.84 ± 0.38	3.98 ± 0.27	1.19	0.3201	0.076	0.714
	L	3.97 ± 0.23	4.19 ± 0.43	4.23 ± 0.27	2.42	0.1066	0.143	0.515
Cerebellum Cortex	R	47.85 ± 4.06	48.79 ± 4.48	49.79 ± 2.72	0.82	0.4510	0.053	0.817
	L	48.55 ± 3.97	49.29 ± 3.90	50.30 ± 3.21	0.68	0.5133	0.045	0.829
Cerebellum White Matter	R	14.33 ± 1.22	14.86 ± 2.14	15.42 ± 1.38	1.51	0.2373	0.094	0.714
	L	14.27 ± 1.12	14.82 ± 1.37	15.59 ± 1.38	3.17	0.0567	0.180	0.411
Brainstem		19.32 ± 1.11	19.64 ± 1.52	20.41 ± 2.34	1.19	0.3188	0.076	0.714

Volume data are shown as mean ± SD and were adjusted for confounding factors including gender, age, scanner, and total intracranial volume using linear regression method. CRPS, patients with complex regional pain syndrome; PAT, patients suffering from chronic pain of other etiologies; CON, control subjects; FDR, false discovery rate; R, right hemisphere; L, left hemisphere.
